# Captive-reared European hamsters follow an offensive strategy during risk-assessment

**DOI:** 10.1371/journal.pone.0210158

**Published:** 2019-01-14

**Authors:** Mathilde L. Tissier, Christophe A. H. Bousquet, Julie Fleitz, Caroline Habold, Odile Petit, Yves Handrich

**Affiliations:** Université de Strasbourg, CNRS, Strasbourg, France; University of Western Australia, AUSTRALIA

## Abstract

Understanding whether captive-reared animals destined to reintroduction are still able to discriminate predators has important implications for conservation biology. The endangered European hamster benefits from conservation programs throughout Europe, in which several thousand individuals are released into the wild every year. Despite this, the anti-predator strategy of hamsters and their ability to maintain predator discrimination in captivity remain to be investigated. Here, we explore the predator discrimination behaviour of captive-reared European hamsters and their response to different predation cues. When first exposed to the urine of cats and goats in a Y-maze test, hamsters spent more time close to the cat scent rather than to the goat scent. In a second experiment, during which hamsters were exposed to a non-mobile European ferret (inside a cage), hamsters significantly increased the time spent close to the ferret’s cage and displayed aggressive behaviour towards the ferret. Furthermore, they did not take refuge inside an anti-predation tube (APT), a device designed to upgrade wildlife underpasses and reconnect wild hamster populations. Finally, when exposed to a mobile ferret (but without physical contact), hamsters displayed mobbing and aggressive behaviours towards the ferret, before taking refuge inside the APT. Taken together, our results show that captive-reared hamsters are still able to detect and react to predation cues, but that they initially adopt an offensive strategy (grunting, spitting, mobbing) during the risk-assessment phase. After risk assessment, however, hamsters used the APT as a refuge. Our study provides important insights into the anti-predator behaviour of hamsters. Testing the efficacy of the APT, a device that will allow upgrading wildlife underpasses for the hamster and other rodents, is also of great importance and is instrumental in conservation efforts for these species.

## Introduction

Predation is a strong selective force that has led prey species to evolve behavioural strategies to minimize predation risk [1]. When exposed to a predation cue, prey species generally display a risk-assessment phase. Depending on the perceived risk [2], animals will freeze (to avoid being spotted by the predator) or display responses that can be characterized as defensive (i.e. fleeing, predator/area avoidance, decreased locomotion and foraging activity or increased vigilance), or more rarely, offensive (the prey species attack or mob the predator). It is generally considered that animals display an offensive response only if freezing or fleeing is not a viable option [3]. Investigating the use of these tactics by a broad diversity of species has been at the base of many studies in behavioural ecology [3–6]. Additionally, understanding the inter-individual variations in the response to predation cues has gained interest in the past decades, given the fitness consequences of such variations [7–9]. In guppies (*Poecilia reticulata*) for instance, individuals approaching their natural predator are less at risk of being attacked than their non-approaching conspecifics [10]. Understanding how individuals perceive and react towards predators is even more important for endangered species for which the anti-predatory strategies are not well understood, which jeopardizes their conservation (Berger et al. 2016). For instance, ensuring that the perception and reaction towards predation cues have not been lost in captive-reared animals [11,12] that are part of restocking programs (in which individuals are released every year to sustain wild populations) appears extremely important.

The European hamster (*Cricetus cricetus*) is one of the most endangered mammals in Europe [13]. Despite this, fundamental studies concerning the behaviour of this species are mostly lacking. Previous studies were descriptive, were conducted in zoological parks [14] and concerned either sexual interactions [15,16] or inter-individual interactions in an urban environment [17]. One aspect of particular importance in the context of current conservation measures is the anti-predator behaviour of hamsters. However, apart from one descriptive study by Eibl-Eibesfeldt [18] that characterized European hamsters as ‘territorial’ and ‘aggressive’, there is currently no information available concerning this aspect. Yet such information is urgently needed since several conservation measures are currently implemented for European hamsters throughout the continent. These measures include restocking programs (with several thousand hamsters released in the fields every year) and the attempt to reconnect wild populations through the improvement of wildlife underpasses. In that context, an ‘Anti-Predation Tube (APT)’ was recently developed [19] to promote the safe-crossing of hamsters and other rodents in wildlife underpasses, through a reduction in predation risk. The features and the use of this device by captive-reared hamsters were tested experimentally under controlled, predator-free conditions [19]. However, its utilization and efficacy have not yet been tested in the presence of a predator.

In the current study, we investigated the perception of predation cues in European hamsters and focused on three main questions: (I) Do captive-reared hamsters perceive predation cues?, (II) Do male and female hamsters respond similarly to controlled predation cues (i.e. defensive versus offensive strategy)? and (III) Does the perception of predation cues affect their decision to take refuge inside the APT? Consequently, we conducted three experimental studies. We first investigated whether captive-reared hamsters are able to discriminate the odour of a predator from that of a non-predator species Such discrimination would confirm the existence of a recognition mechanism [20]. We used a Y-maze test, with the urine of a predator (domestic cat, *Felis silvestris catus*) placed on one branch of the maze and the urine of an herbivore (goat, *Capra hircus*) placed on its opposite side. We predicted that hamsters would avoid the branch with the predator urine even display escape behaviour, as has been observed in most rodents and lagomorphs tested so far [4,20–22]. In a second experiment, we evaluated the use of the APT in the presence of a ‘non-mobile’ (i.e. caged) predator, the European ferret (*Mustela putorius furo*). We specifically investigated how hamsters reacted to the presence of the ferret, and whether its presence led to an utilization of the APT. We expected that hamsters would avoid the ferret [4] and increase the utilization of the APT. In a third experiment, we tried to simulate a more natural situation by using the same set-up, albeit with a mobile ferret (i.e. physical contact with the hamster was prevented). In addition, we fed the ferret with corpses of European hamsters before trials, expecting a stronger behavioural reaction [5] and a further increase in APT utilization.

## Methodology

### Hamsters and housing conditions

European hamsters are among the largest hamster species in the world [23]. Males and females weigh on average 350 g and 250 g, respectively, with important seasonal and local variations [23,24]. Hamsters are omnivorous and feed on seeds, roots, green parts of plants, invertebrates and small vertebrates [23,25]. This species is described as territorial and very aggressive [14,18,23,26], although no studies have investigated the behavioural responses of hamsters towards predators. Adult European hamsters are predated by red foxes (*Vulpes vulpes*), stoats (*Mustela erminea*), birds of prey (e.g. common buzzard *Buteo buteo*), domestic cats, badgers (*Meles meles*) and dogs (*Canis lupus f*. *familiaris*) [27,28]. Juveniles are also predated by common kestrels (*Falco tinnunculus*), long-eared owls (*Asio otus*), grey herons (*Ardea cinerea*), crows (*Corvus corone corone*) and rooks (*Corvus frugilegus*) [27]. In the French distribution area of the European hamster, a video monitoring of wildlife underpasses showed that cats, ferrets and foxes impose a high predation risk [29]. Since hamsters are strongly territorial, hamsters, we housed them in single transparent Plexiglas cages (420*265*180 mm, D*W*H) before and after experiments. We used wood and shredded paper to enrich their environment. Animals were provided with an *ad libitum* supply of water and food pellets (N° 105, from Safe, Augy, France).

### Ethics statement

The experimental protocols followed the EU Directive 2010/63/EU and the guidelines for animal experiments and the care and use of laboratory animals, and were approved by the Ethical Committee (CREMEAS) under the agreement number 02015033110486252 (A PA FIS#397). 01.

### The Y-maze test

**Fig 1:**
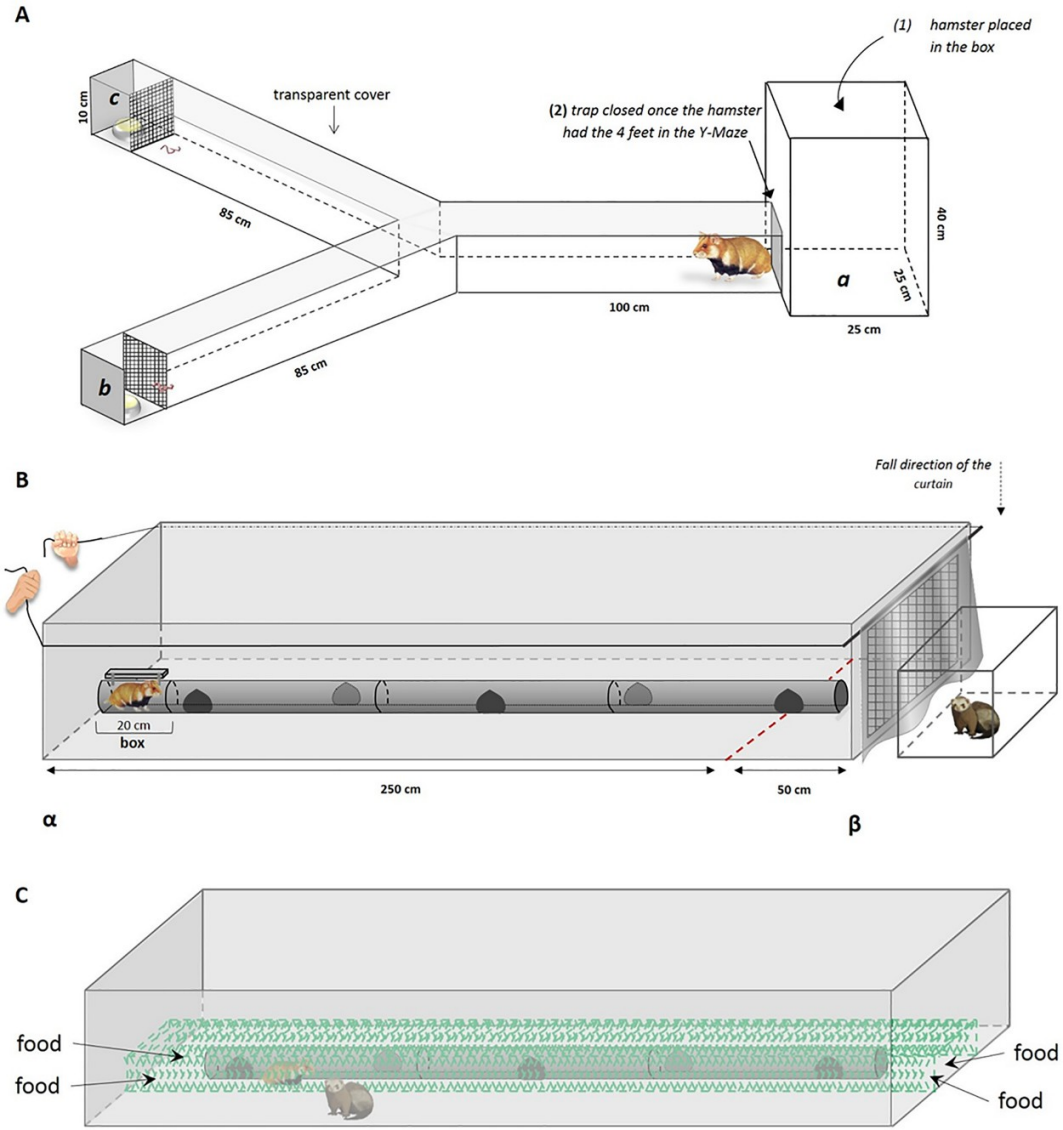
Schematic representation of the experimental designs for (A) the Y-Maze test, (B) the APT efficacy test with a non-mobile ferret and (C) the APT efficacy test with a mobile ferret. The associated methodology is described in the text, sections ‘*The Y maze test*’, ‘*The APT efficacy test with a non-mobile ferret*’ and ‘*The APT efficacy test with a mobile ferret*’.

In April-May 2014, 9 European hamsters (5 ♂ and 4 ♀) were confronted with a forced choice situation (Y-maze test). One branch of the maze contained a predator odour (urine from 15 adult non-neutered female domestic cats), while the other branch contained a non-predator odour (urine from 5 adult, non-neutered female domestic goats). The cat urine was collected by a veterinarian before the neutering procedure. Goat urine was collected during natural urination. Within the hour of urine collection, the urine from all cats was mixed to obtain a homogenous sample. Goat urine was treated in the same way. We then prepared compresses (STERILUX ES 7,5*7,5 cm; CENTRAVET) with 1 mL of either cat or goat urine, which were immediately frozen at -28°C. One day before experimental trials, each hamster was acclimated to the maze for 5 minutes. During these trials, compresses with 1 mL of water instead of urine were used. On experimentation day, compresses with cat and goat urine were thawed15 minutes prior to each experiment. For thawing, compresses were placed inside two separated, closed Petri dishes at T_a_ = 22°C.The Petri dishes with cat and goat urine were placed either into the ***b-branch*** or the ***c-branch*** of the Y-maze. They were placed behind a metal grid (Fig 1A) and were both opened ~3min before the start of a trial. The maze was closed with a transparent plastic lid, so that the cat and goat odour could diffuse into the respective branches. Approximately 2g of earthworms (*Lumbricus terrestris*), an appetent food for the hamster, were placed close to the grids (see Fig 1A). Each hamster was placed in a box at the ***a-branch*** of the maze. Once a hamster left the box, the latter was closed and the 5min recording started (hamsters were filmed from the top). In our video analysis, we recorded the time a hamster spent within each branch of the maze, how frequent each branch was visited, as well as the occurrence of body-shaking (or snorting) episodes within branches as a measure of disturbance [30].

To control for potential effects of urine position (b- versus c-branch of the maze) and time of day, each hamster was tested four times (with one day of interval each test) as follows: (i) cat urine on the right and goat urine on the left, (ii) cat urine on the left and goat urine on the right, (iii) in the morning and (iv) in the afternoon. Test order was randomized and the maze was cleaned with ethanol (70%) and aired for 7 minutes between trials, during which time the compresses and worms were renewed. All the tests (36 in total) were carried out in low-light conditions (20W-light bulb) in a room at T_a_ = 17±1°C. A technical problem with the camera prevented filming during one trial, leaving a sample size of 35 recorded trials (for a total of 175 minutes).

### The APT efficacy test with a non-mobile ferret

Experiments were conducted in April-May 2014 with a total of 8 hamsters (4 ♂ and 4 ♀), which were all tested once in four different conditions (see below), resulting in a total of 32 trials (328 min of recording). Our set-up consisted of a rectangular PVC enclosure and an anti-predation tube (APT) in its centre (Fig 1B). The PVC enclosure (3*1*0.4m, length*width*height) had a metal grid (wire mesh: 25*25 mm) at one extremity and resembled the typical shape used for French wildlife underpasses [29]. The prototype of an APT, 2.78m long, previously developed to promote the safe crossing of small mammals within wildlife underpasses and culverts [19], was placed in the centre of the PVC enclosure. The APT consisted of a PVC tube (10cm in diameter) with lateral openings at 1m intervals on both sides, enabling hamsters to rapidly enter and exit the APT from anywhere within the PVC enclosure (Fig 1B). An opaque curtain masked the grid at the extremity of the enclosure. Behind this curtain (i.e. outside the PVC enclosure) and before a trial, the experimenter placed a cage (0.8*0.4*0.4m) containing a female European ferret (extremity β, Fig 1B).At the beginning of a trial, a hamster was released at the extremity α of the PVC enclosure. Hamsters typically started to explore the PVC enclosure and when they first crossed a line marking a distance of 50cm to the metal grid, the curtain hiding the ferret was smoothly lowered by the experimenter (Fig 1B), revealing the ferret to the hamster. Hamster behaviour was filmed for the 5 min that a trial lasted. During subsequent video analysis, we recorded both the time each hamster spent inside the APT and the time it spent within 50cm of the ferret cage. We also recorded the numbers of ‘U-turns’ (i.e., when a hamster quickly turned away from the ferret cage and moved towards the opposite end of the enclosure). Furthermore, we recorded exploratory behaviours: the frequency of scraping (when an individual scratched the floor) and rearing (when the individual raised the upper part of the body), as well as agonistic behaviours, i.e. spitting, grunting and posture attack (see [14]). After each trial, the room was aired out and the APT, the enclosure and the ferret cage were cleaned with ethanol (70%). Between trials, the ferret was kept in a cage with hay, litter, water and food *ad libitum*. Two hours before each session, its food was removed. Before predator trials, all hamsters were tested under three ‘predator-free’ conditions to validate their spontaneous use of the set-up: hamsters were either placed to the left of the APT, to the right of the APT or inside the APT. Trial duration was 5 min each. Since the initial position of a hamster had no effect on their use of the set-up (see [19] for detailed results) and since the order of the three predator-free conditions was randomized, results from these trials were pooled to create a control condition (‘predator-free’, P_free_), to which the predation P condition was compared. All experiments were carried out in low-light conditions at T_a_ = 21±2°C.

### The APT efficacy test with a mobile ferret

Trials were conducted in April-May 2016 with 16 hamsters (6 ♂ and 10 ♀). We used the set-up described above, albeit, with some modifications. First, the male ferret used in these trials was placed inside the PVC enclosure (Fig 1C). To ensure the safety of both the hamster and the ferret, while allowing for physical proximity and potential predatory attempts from the ferret, we built a structural separation. A rigid metal grid with a small mesh size (25*25mm) was placed alongside the APT, effectively separating hamster and ferret (Fig 1C). Dimensions of the inner grid enclosing the APT were 3*0.5*0.12m (Fig 1C). Accordingly, both the hamster and the ferret could see, smell and move towards each other, up to a close distance but physical contact was avoided. After introduction of both at the beginning of a trial, two metal grids (1.6*1.1m each) were used to cover the PVC enclosure, preventing the ferret from escaping.

In these trials with a mobile predator, we wanted to maintain the motivation of hamsters to feed and/or hoard food, while they should also perceive a high level of predation risk. Hence, we food deprived hamsters from the evening preceding a trial and we added food rewards in all four corners of the inner metal grid. The food rewards consisted of 10 pumpkin seeds and ¼ of a carrot slice, placed directly on the floor. Our protocol included two conditions (‘predator condition’ versus ‘predator-free condition’), each consisting of three phases. In the ‘predator’ condition (P), the hamster was first introduced to the inner part of the apparatus (surrounded by the inner metal grid, Fig 1C) for 10min (pre-treatment phase). Then, in a second step, the ferret was added for 5min (treatment phase with ferret). Thereafter, the experimenter entered the room, and removed the ferret from the apparatus, leaving the hamster for an additional 10min within the apparatus (post-treatment phase). In the ‘predator-free’ condition (P_free_), the hamster was introduced to the inner part of the apparatus for 10min (pre-treatment phase). Then, the experimenter opened the upper metal grid and mimicked the introduction of the ferret, without actually placing the ferret inside. Five minutes after this ‘fake introduction’ (treatment phase without ferret), the experimenter entered the room, opened the upper metal grid, mimicked the removal of the ferret and left the hamster for a further 10min inside the apparatus (post-treatment phase). Each hamster participated in trials with both conditions, with half of the individuals passing the P condition in their first session (and the P_free_ condition in their second session) and the other half passing the P_free_ condition in their first session (and the P condition in their second session). Trials with the two conditions were separated by one week. All trials were filmed. During subsequent video analysis, we recorded the time hamsters spent in the centre versus the two ends of the apparatus and also estimated their use of the APT. For this, we counted the number of entries into the APT, the time spent inside the APT, and the distance travelled within the APT (distance between entry and exit holes). Food consumption of hamsters was documented by noting the number of eaten/hoarded pumpkin seeds and carrot slices, and also by noting the duration hamsters engaged in eating/hoarding food. Finally, we recorded the number of mobbing events (when hamsters approached, harassed and sometimes even attacked the ferret), to evaluate whether hamsters used an offensive or defensive strategy.

If a hamster consumed food during any of the three trial phases, we replenished the food rewards, so that 10 pumpkin seeds and a quarter of a carrot slice were always present at the beginning of each phase. Thus, the maximal reward a hamster could consume was 3*4*10 = 120 pumpkin seeds and 3*4*1 = 12 quarters of carrot slices. All hamsters participating in the ‘mobile ferret’ trials were new to the apparatus (i.e. they had not participated in the ‘non-mobile ferret’ trials). To motivate the ferret, it was fed every morning with approximately 50g of hamster corpse (died of natural causes in our captive breeding unit). Feeding took place every morning between 8:00 and 8:30 am, except on experimental days, when the ferret was fed after the end of experimentation.

### Data analyses

During the Y-maze trials, the *cumulative time spent in each branch* of the maze (in seconds) was normally distributed and was therefore analysed using a Linear Mixed Model (LMM). The *number of body-shaking* and the *number of visits in each branch* of the Y-maze were count variables and were therefore analysed using Generalized Linear Mixed Models (GLMM) with a Poisson probability distribution and a log link function. We included the type of odour, the sex, the order of the trial, the side where the predator odour was presented and the sex*odour interaction as fixed factors. The identity of the individuals was included as a random factor to control for repeated measures on the same individual. Given the reduced sample sizes of our experiments, we first started with the most parsimonious model including the sex and condition and used an ascendant stepwise approach to include other variables of interest (i.e. the sex*condition interaction, the order of the trial to test for an eventual habituation of the hamster and the side where the odour was presented for the Y-maze test, to control for a laterality bias). For LMMs, final model selection was based on the best AICc (Akaike information criterion corrected for small samples) value.

Regarding the APT efficacy test with a non-mobile ferret: given that the P_free_ test lasted 12 min while the P test lasted 5 min (to avoid a habituation of the hamster or the ferret), all the variables were analysed per unit of time (i.e. per min). We looked at the effect of the condition (P_free_ or P) and the sex on the *proportion of time spent inside the APT*, the *proportion of time spent behind the 50cm line*, the *U-turn frequency* from the ferret extremity towards the opposite side, the *scraping frequency* and the *rearing frequency* using the non-parametric Wilcoxon matched-pairs signed rank test.

For the APT efficacy test with a mobile ferret, we first characterised each hamster according to its behavioural reaction to the presence of the ferret. During the treatment phase of the P condition (i.e. when the ferret was present), we noted the number of mobbing events displayed by the hamster towards the ferret and how many times the hamster entered the APT. Each hamster that used the APT at least once or did not mob the ferret was classified as “cautious” (8 hamsters), while each hamster that mobbed the ferret at least once and did not enter the APT during this phase was classified as “mobbing” (8 hamsters). We then analysed the number of mobbing events using a GLM (probability distribution: Poisson, link function: log), with the sex of the hamster as a fixed factor. Secondly, we measured the seven following variables in the pre-treatment phase and in the post-treatment phase: (i) *time spent inside the APT*, (ii) *time spent in the middle of the apparatus*, (iii) *time spent in the corners of the apparatus*, (iv) *number of entries into the APT*, (v) *distance travelled within the APT*, (vi) *time spent eating/hoarding*, and (vii) *number of eaten/hoarded pumpkin seeds and carrot slices*. The distributions of the variables (i) to (iii) did not differ from the normal distribution (normality was assessed via a Shapiro-Wilk test). Therefore, these variables were analysed using LMMs, with the lmer() function from the lme4 package [31]. The variables (iv) and (v) followed a Poisson distribution, while the variable (vi) followed a negative binomial distribution. Finally, the variable (vii) followed a binomial structure because for each phase we knew the proportion of eaten/hoarded food items. Models (iv) to (vii) were implemented with the mixed() function from the afex package [32], by setting the appropriate “family” argument (poisson, negative binomial with θ = 3 and binomial, respectively). For each model, we included the experimental period (pre- or post-treatment), the condition (P or P_free_), the behavioural type of the hamster (cautious or mobbing), the sex of the hamster and the session number (first or second), as well as all two-way interactions involving the presence of the ferret, as fixed factors. We included these interactions because we expected that the presence of the ferret may affect (i) one sex more than the other, (ii) cautious hamsters more than mobbing hamsters, (iii) one session more than the other and, more importantly, (iv) the post-treatment period rather than the pre-treatment period. The identity of the hamsters was included as a random factor for repeated measures on the same individual. For this analysis, we present estimated marginal means computed using the emmeans R package [33].

For all models, key explanatory variables (sex, condition and sex*condition interaction) were targeted based on a study in the golden hamster (*Mesocricetus auratus*), which showed that males and females display differential responses to predation cues [34] and a study describing females hamster as more aggressive than males [18]. Data presented are means ± SEM. Normality of the residuals of every model was tested using a Kolmogorov-Smirnov test or a Shapiro-Wilk test. Analyses were conducted using R (R-3.2.3) with the RStudio interface (RStudio, Inc., 0.99.491.0), and the significance threshold was set at p < 0.05. Figures were prepared using GraphPad prism software (Version 5, La Jolla, USA) or the R package ggplot2 (ggplot2.org)[35].

## Results

### The Y-maze test

**Fig 2:**
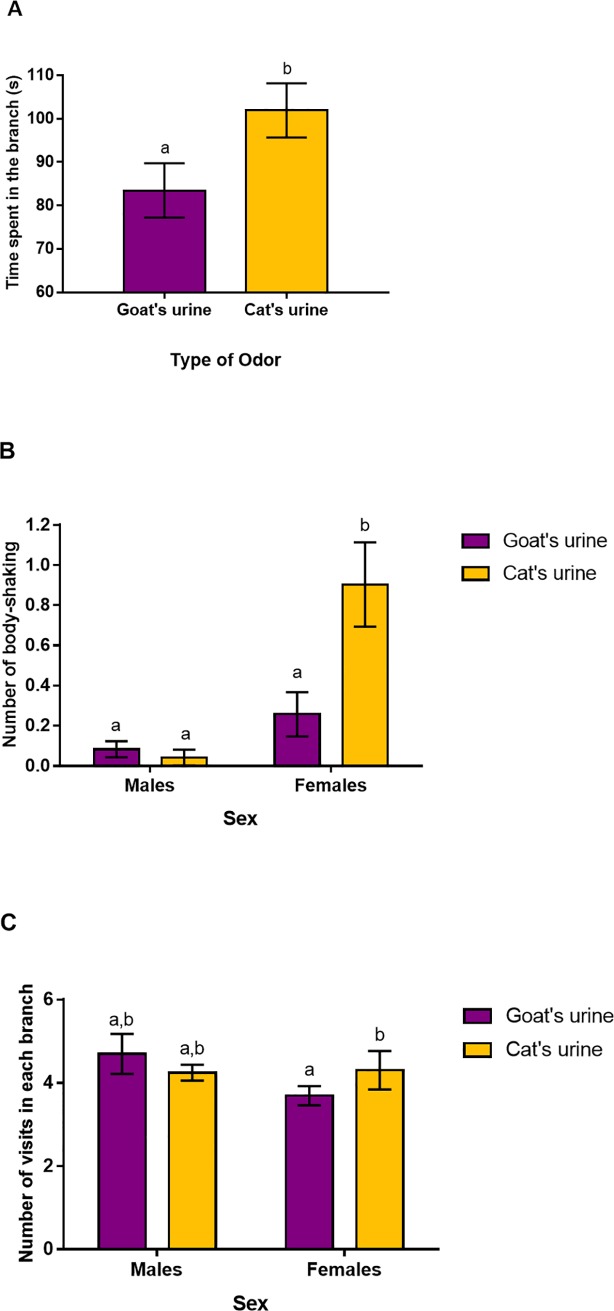
Effects of the type of odour (cat or goat urine) on (A) the time spent in each branch, (B) the mean number of body-shaking episodes and (C) the number of visits in each branch of the Y-maze. In (B) and (C), mean ± SE are represented according to the sex and the type of odour (because of the significant sex*odour interaction). Different letters mean significant differences between the groups. In (C), we found no differences regarding the number of visits in each branch between males and females exposed to goat urine and females exposed to cat urine (p = 0.055 and p = 0.058, respectively).

The 9 individuals did not show any side bias: the right and left branches were chosen first on 17 and 18 occasions, respectively. We thus did not use the position of the odour in the analyses of subsequent experiments. We found an effect of the type of odour on the *time spent in each branch* (F_1,58_ = 4.37, p = 0.041): hamsters spent significantly more time in the branch with the cat urine than in the branch with the goat urine (Fig 2A, 101.9±6.3s and 85.5±6.3s for cat and goat scents, respectively). We found no effect of the other variables or the interaction on the time spent in each branch, including the order of the trial that had no significant effect on any of the response variables (p > 0.1). Regarding the *number of body-shaking episodes*, we found no effect of the type of odour (Wald χ^2^ = 0.5, p = 0.5) but an effect of the sex (Wald χ^2^ = 8.5, p = 0.003) and the sex*odour interaction (Wald χ^2^ = 6.1, p = 0.014). Females displayed a higher mean number of body-shaking episodes (Fig 2B) when faced towards the cat urine than when faced towards the goat urine (mean difference = -0.65±0.18, p < 0.001). No differences were found for males between the two odours (Fig 2B, mean difference = 0.04±0.04, p = 0.28). No effects of the other variables were found on the number of body-shaking episodes (p > 0.1). Finally, regarding the number of visits in each branch, we found no effect of the odour (Wald χ^2^ = 0.4, p = 0.6) nor the sex (Wald χ^2^ = 1.1, p = 0.3) but an effect of the sex*odour interaction (Fig 2C, Wald χ^2^ = 7.96, p = 0.005). Females visited the branch with the cat urine significantly more often than the branch with the goat urine (4.3±0.4 and 3.7±0.2 times, respectively; p = 0.035). We found no differences in the number of visits between the two odours for males (which visited the cat and the goat branches on average 4.2±0.2 and 4.7±0.5 times, respectively; p = 0.16).

### The APT efficacy test with a non-mobile ferret

When the ferret was present, hamsters displayed a higher *U-turn frequency* (Table 1; Wilcoxon signed rank test, T = 27, p = 0.03, N = 8), a lower *rearing frequency* (Table 1; T = 0, p = 0.02, N = 8), and a lower *scraping frequency* (Table 1; T = 0, p = 0.03, N = 8). They also spent a higher *proportion of time behind the 50cm line* (Table 1; T = 27, p = 0.03, N = 8). However, we found no differences between the P and the P_free_ conditions when considering the *body-shaking frequency* (Table 1; T = 0, p = 0.2, N = 8) and the *proportion of time spent in the APT* (Table 1; T = 13, p = 0.9, N = 8). In the P condition, agonistic behaviours (spitting, grunting and posture attack [14]) were recorded in 3 of the 4 males, whereas these behaviours were never observed in the P_free_ conditions. However, this difference was not significant (Table 1; T = 6, p = 0.2, N = 8).

**Table 1:** Effects of the presence of the predator on hamster behaviour in the APT efficacy test with a non-mobile ferret.

Variable	Condition	Mean±SE	Predator effect
**U-turn frequency (no/min)**	**P**_**free**_	**0.01 ± 0.01**	**+**
	**P**	**0.28 ± 0.08**	
**Rearing frequency (no/min)**	**P**_**free**_	**0.63 ± 0.08**	**-**
	**P**	**0.23 ± 0.07**	
**Scraping frequency (no/min)**	**P**_**free**_	**0.07 ± 0.02**	**-**
	**P**	**0**	
**Proportion of time spent beyond the 50cm limit (s/min)**	**P**_**free**_	**0.30 ± 0.03**	**+**
	**P**	**0.51 ± 0.09**	
**Body-shaking frequency (no/min)**	P_free_	0.05 ± 0.02	NE
	P	0.09 ± 0.04	
**Proportion of time spent in the tube (s/min)**	**P**_**free**_	0.05 ± 0.01	NE
	**P**	0.06 ± 0.04	
**Agonistic behaviour frequency (no/min)**	**P**_**free**_	0	NE
	**P**	0.1 ± 0.05	

Mean±SE are presented for the Predator condition (P) and the Predator-free condition (P_free_). Means in bold represent significant differences between the two conditions (Wilcoxon signed rank test). The + and—indicate the direction of the difference when significant, and NE indicates “No effects”. See the Methodology for details and the Results section for statistics.

Compared to females, males displayed a higher *U-turn frequency* (Wald *χ*^2^ = 5.5, df = 1; p = 0.02; ♀ = 0.30±0.03 and ♂ = 0.41±0.05U-turns.min^-1^) and spent a higher *proportion of time in the APT (*Wald *χ*^2^ = 5.2, df = 1, p = 0.023; ♀ = 0.93±0.25 and ♂ = 0.48±0.06s.min^-1^). However, sex did not influence the *proportion of time spent behind the 50cm line* (Wald *χ*^2^ = 3.7, df = 1, p = 0.055; ♀ = 0.10±0.01 and ♂ = 0.17±0.02s.min^-1^) or the three other variables (p > 0.5).

### The APT efficacy test with a mobile ferret

**Fig 3:**
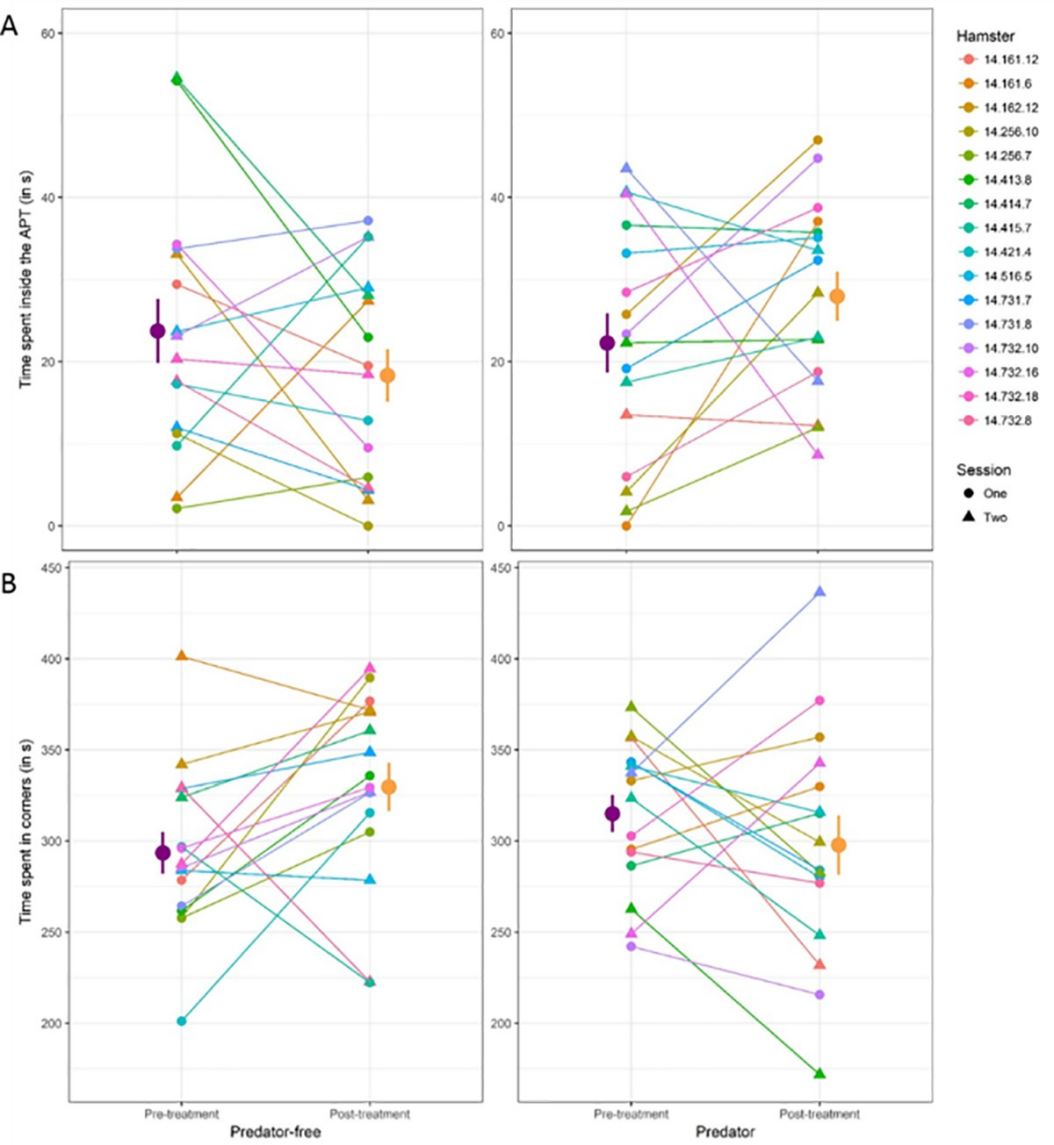
Effects of the presence (Predator, “P”) or absence (Predator-free, “P_free_”) of the ferret during the 5-min treatment phase. The effect of the presence of the predator on (A) the time spent inside the APT and (B) the time spent at the two ends of the apparatus are presented. Orange circles and error bars represent the mean and its associated standard error for the P condition. Purple circles and error bars represent the mean and its associated standard error for the P_free_ condition. Coloured circles and lines are individual data of the 16 hamsters used in the experiment. Individual identities are reported on the right: the first number (e.g. 14) indicates the birth year; the second number indicates the litter identity and the third number indicates the identity of the individual. Circles indicate that the corresponding condition occurred during the first session and triangles indicate that the corresponding condition occurred during the second session.

After being confronted with a mobile ferret, hamsters spent more time inside the APT during the post-treatment phase when compared with the pre-treatment phase (+5.7±4.1s), which was not the case in the control condition (-5.4±4.1s). This interaction was significant (Fig 3A, F_1;64_ = 4.39, p = 0.04). Independently from this effect, sex also played a role: females spent more time in the APT than males (+15.4±3.0s, F_1,64_ = 31.7, p < 0.001). Besides, after being confronted with a mobile ferret, hamsters decreased the time spent at the two ends of the apparatus in the post-treatment phase, when compared with the pre-treatment phase (-17.3±17.0s), whereas hamsters did the contrary in the control condition (+36.2±17.0s). This interaction is significant (Fig 3C, F_1;48_ = 5.69, p = 0.02). As a corollary, after being exposed to a mobile ferret, hamsters tended to spend more time in the middle of the apparatus in the post-treatment phase, when compared with the pre-treatment phase (+11.7±17.0s), whereas hamsters did the contrary in the control condition (-30.9±17.0s). However, this interaction did not reach significance (F_1,48_ = 3.56, p = 0.07). Means±SEM and a summary of these results are presented in Table 2.

**Table 2:** Effects of the presence of the predator on hamster behaviour in the APT efficacy test with a mobile ferret.

Variable	Condition	Post-treatment—Pre-treatment	Significance	Predator effect
		(Mean ± SE)		
Time spent in tube (s)	P_free_	**-5.4 ± 4.1**	**F**_**1,64**_ **= 4.39**	+
	P	**+5.7 ± 4.1**	**p = 0.04**	
Time spent at extreme ends (s)	P_free_	**+36.2 ± 17.3**	**F**_**1,48**_ **= 5.69**	-
	P	**-17.3 ± 17.3**	**p = 0.02**	
Time spent in the centre (s)	P_free_	-30.9 ± 17.0	F_1,48_ = 3.6	NE
	P	+11.7 ± 17.0	p = 0.07	
Variable	Condition	Post-treatment / Pre-treatment	Significance	Predator effect
		(Ratio ± SE)		
Number of entries in tube	P_free_	1.06 ± 0.16	χ^2^_1,10_ = 2.51	NE
	P	1.49 ± 0.23	p = 0.11	
Distance traveled in tube (cm)	P_free_	**1.01 ± 0.02**	**χ**^**2**^_**1,10**_ **= 357.56**	+
	P	**1.60 ± 0.03**	**p < 0.001**	
Time spent eating (s)	P_free_	0.75 ± 0.18	χ^2^_1,11_ = 0.62	NE
	P	0.58 ± 0.14	p = 0.43	
Number of consumed food items	P_free_	**2.13 ± 0.28**	**χ**^**2**^_**1,10**_ **= 50.53**	-
	P	**0.57 ± 0.07**	**p < 0.001**	

Mean±SE or Ratio ±SE are presented according to the Predator condition (P) and to the Predator-free condition (P_free_). The + and—indicate the direction of the difference when significant and NE indicates “No effects”. See the Methodology for details and the Results section for statistics.

In general, females entered the APT more often than males (females: 6.99±0.42 entries, males: 2.73±0.34 entries, ratio: 2.56±0.35; χ^2^_1,10_ = 26.37, p < 0.001). Furthermore, hamsters entered more often the APT in the post-treatment phase than in the pre-treatment phase (post-treatment: 4.89±0.41 entries, pre-treatment: 3.90±0.36 entries, ratio: 1.25±0.14; χ^2^_1,10_ = 4.39, p < 0.05). The exposure to a mobile ferret induced hamsters to use the APT for longer distances in the post-treatment phase, when compared with the pre-treatment phase (post-treatment: 384.3±29.7 cm, pre-treatment: 240.3±18.7 cm, ratio: 1.60±0.03). This was not the case for the P_free_ condition (post-treatment: 303.9±23.5 cm, pre-treatment: 299.9±23.2 cm, ratio: 1.01±0.02). This interaction was highly significant (Table 2, χ^2^_1,10_ = 357.6, p < 0.001). Once in the APT, females travelled longer distances than males in the P_free_ condition (females: 553.5±51.8 cm, males: 164.6±20.1 cm, ratio: 3.36±0.52) and even more so in the P condition (females: 585.6±54.8 cm, males: 157.7±19.2 cm, ratio: 3.71±0.57). This interaction was significant (χ^2^_1,10_ = 9.1, p < 0.01). Similarly, hamsters that exhibited a cautious behavioural response to the presence of the ferret travelled longer distances in the APT than “mobbing” hamsters in the P_free_ condition (cautious: 321.6±34.4 cm, mobbing: 283.4±30.3 cm, ratio: 1.13±0.17) and even more so in the P condition (cautious: 360.9±38.5 cm, mobbing: 255.8±27.4 cm, ratio: 1.41±0.21). This interaction was again significant (χ^2^_1,10_ = 81.9, p < 0.001). Finally, hamsters travelled longer distances inside the APT in their first session (irrespective of whether it was the P or the P_free_ condition), when compared with their second session (first: 307.9±23.6 cm, second: 297.9±22.9 cm, ratio: 1.03±0.01; χ^2^_1,10_ = 7.67, p < 0.01).

In the P_free_ condition, females spent more time eating than males (females: 36.0±6.9 s, males: 27.8±6.9 s, ratio: 1.30±0.41). Contrarily, in the P condition, males spent more time eating than females (females: 32.2±6.1 s, males: 48.9±11.8 s, ratio: 0.66±0.20). This interaction was significant (χ^2^_1,11_ = 4.23, p < 0.05). Additionally, hamsters spent more time eating in their second session (first: 23.0±3.5 s, second: 54.6±8.5 s, ratio: 0.42±0.07; χ^2^_1,11_ = 28.73, p < 0.001), when they behaved cautiously (cautious: 51.7±9.4 s, mobbing: 24.2±4.6 s, ratio: 2.13±0.55; χ^2^_1,11_ = 7.05, p < 0.01) and during the pre-treatment phase (post-treatment: 28.8±4.6 s, pre-treatment: 43.6±6.8 s, ratio: 0.66±0.11; χ^2^_1,11_ = 6.02, p < 0.05). The presence of the ferret modified the foraging strategy of the hamsters. Indeed, in the P_free_ condition, hamsters foraged more successfully in the post-treatment phase (post-treatment: 70.3±6.7%, pre-treatment: 52.6±7.9%, odds ratio: 2.14±0.28), whereas in the P condition, hamsters foraged more successfully in the pre-treatment phase (post-treatment: 56.4±7.8%, pre-treatment: 69.3±6.8%, odds ratio: 0.57±0.07). This interaction was significant (χ^2^_1,10_ = 50.5, p < 0.001). Additionally, the P_free_ condition favoured the foraging success of cautious hamsters (cautious: 66.1±9.7%, mobbing: 57.5±10.6%, odds ratio: 1.44±0.87), whereas the P condition favoured the foraging success of mobbing hamsters (cautious: 54.5±10.7%, mobbing: 70.9±9.0%, odds ratio: 0.49±0.30). Again, this interaction is significant (χ^2^_1,10_ = 32.3, p < 0.001). Finally, foraging success was significantly better in the second session than in the first session (first: 59.7±7.5%, second: 65.2±7.1%, ratio: 0.79±0.07; χ^2^_1,10_ = 6.28, p < 0.05).

**Fig 4:**
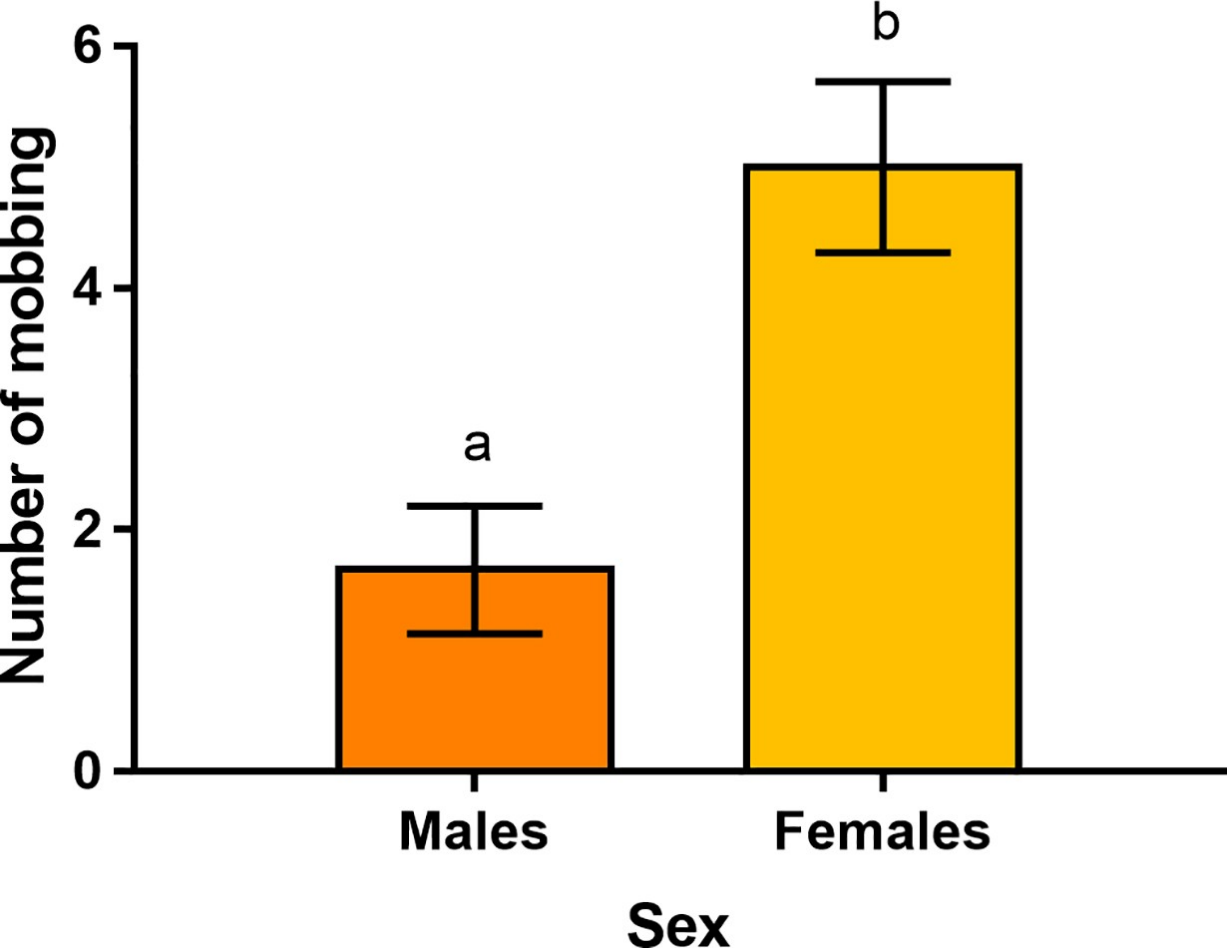
Frequency of mobbing behaviour displayed by hamsters in the APT efficacy test with a mobile ferret according to sex. Males are presented in dark orange, while females are presented in light orange. Mobbing behaviour includes running towards the predator, grunting, spitting and direct attack.

Within the 5 minutes of the ferret presence, 11 out of 16 hamsters displayed mobbing behaviours towards this predator. We found that, on average, females mobbed the ferret significantly more often than males (females: 5.0±0.7mobbing, males: 1.7±0.5mobbing; Wald χ^2^ = 10.1, p = 0.002, N = 16; Fig 4).

## Discussion

Contrary to our initial predictions, captive European hamsters did not flee when confronted with the odour of one of their known predators (i.e. cat odour) or with the predator itself (i.e. the European ferret). Rather, they significantly increased the time spent near the cat odour or the ferret, while displaying disruptive (body-shaking) or aggressive behaviour. Therefore, European hamsters seem to display a bold personality type [7], as they did not avoid areas or immediately fled, when confronted with cues from some of their natural predators. This contrasts with observations from mice, voles, rabbits, golden hamsters and rats [4]. The increased time spent inside the APT after exposure to a mobile ferret indicates that European hamsters adopt more cautious behaviours, once predator cues have disappeared.

### Y-maze test

The results of this test indicate that predator-naïve hamsters are able to discriminate the urine of a cat (the predator model in our study) from the urine of a goat (the neutral scent in our study). Yet they spent more time close to the cat odour than to the goat odour, which would seem counter-intuitive, based on previous studies concerning the reaction of rodent prey-species to predator odours [4,20,21]. However, females displayed a greater number of body-shaking episodes when facing cat urine than when facing goat urine. This is a characteristic reaction to disturbance found in rodents, and is usually interpreted as agonistic behaviour [30,36]. Still, in our case, the disturbance presented by the cat urine may not have been maximal, which could explain these bold behaviours. Indeed, it has been shown that a predator’s diet influences the perception and strength of reactions in dwarf hamsters, when confronted with predator urine [5]. Hence, it is conceivable that European hamsters might have reacted differently to the cat urine, if these cats had been fed with hamsters before urine collection.

Furthermore, the reaction of prey species to predators appears to be species dependent [4]. Most prey species–from mice to ungulates–display a ‘defensive strategy’ (escape behaviour, decreased locomotion, predator or area avoidance…) [4,37,38]. However, some species display an ‘offensive strategy’ (aggressive behaviours, mobbing and no direct escape) in response to a first exposure to predation cues [3]. Taken together, the results of the Y-maze test suggest that European hamsters rather use an offensive strategy, when compared with other rodents that generally show predator-urine avoidance [4,20,21]. To confirm such a different strategy in European hamsters, tests with different, ecologically-relevant predators (e.g. foxes, badgers) and non-predators (e.g. hares, rabbits) are needed.

### The APT efficacy test with a non-mobile ferret

The results of this test, revealing significant behavioural differences between the ‘Predator’ condition in the presence of the European ferret and the ‘Predator-free’ condition, indicate that captive-reared hamsters perceived the presence of this predator species. However, contrary to our predictions, hamsters spent more time beyond the 50cm line (i.e. close to the predator) in the P condition, when compared with the P_free_ condition. Since we used a different predator species (i.e. the European ferret) in these tests, it reinforces the results of the Y-maze test with respect to the use of an ‘offensive strategy’ against predators in European hamsters. In parallel, we observed a behavioural shift in hamsters in the presence of the ferret: exploratory (rearing) and grooming behaviours were significantly decreased or even suppressed in favour of U-turns (significantly increased in the P condition) and agonistic behaviours (recorded only in males). When considered by itself, the increased number of U-turns in the presence of the predator would indicate an avoidance behaviour. However, if we consider this greater number of U-turns in association with the increased amount of time spent close to the predator, this rather suggests a ‘risk assessment’ phase [39], which precedes decision-making (staying or escaping and taking refuge in a secure area) [40]. Nonetheless, hamsters did not increase their use of the APT in the presence of the predator, which could be explained in several ways. First, hamsters may not have perceived the ferret as a real predator (after a phase of risk assessment) but rather displayed an attraction for novelty [41]. To control for this, it would be important to test the hamsters with a non-predator species. Yet, although possible, this explanation is unlikely, given the aggressive behaviours displayed by three of the four males and the suppression of exploratory/grooming behaviours in all hamsters. However, the ferret’s presence might not have represented an immediate risk of predation, strong enough to push hamsters to use the APT. Indeed, since the ferret was in a small cage, it was limited in its movements and was consequently fairly inactive. Moreover, hamsters were separated from the ferret by two layers (metal grid and cage), which might have prevented defensive responses by the hamsters. Finally, exposure to predators may have long-lasting effects and induce predator avoidance in prey species, once the direct presence of the predator is no longer perceived [3,42].

### The APT efficacy test with a mobile ferret

The test with the mobile ferret confirms that captive European hamsters are using an offensive strategy towards a predator. Hamsters mainly countered the direct threat of the predator by mobbing it. However, some hamsters also used the APT to protect themselves from the predator’s presence. We therefore observed two different strategies in the presence of the predator: a defensive, cautious strategy (use of the APT in the presence of a mobile ferret or no aggression, 8 hamsters) and an offensive, mobbing strategy (mobbing the predator without taking refuge, 8 hamsters). Results of this third experiment also show that captive-reared hamsters displayed lasting behavioural changes when exposed to a mobile predator that was previously fed with hamster corpses. Indeed, hamsters used the APT more frequently and spent more time within the APT after predator exposure. Hamsters also avoided the extreme ends of the apparatus, which were the furthest away from the APT and contained food. However, the presence of a mobile predator did not significantly affect the time hamsters spent foraging (i.e. the time spent on the food rewards). Yet hamster foraging success (i.e. the number of eaten or hoarded food items) was lower when exposed to a predator, and this was particularly true for cautious individuals. Given that each hamster was tested only once with the ferret, we cannot assess whether the strategy a hamster uses is repeatable over time. It is possible that the choice of strategy depends on subtle cues delivered by the ferret on its immediate capture intentions [2].

### Offensive strategy and ecological implications

Taken together, the results of our three experiments show that European hamsters display several signs of risk evaluation and bold behaviours before escaping cat urine or the presence of the ferret. Many individuals even displayed mobbing behaviour (or even direct attacks) towards the predator, revealing that they adopted an offensive strategy. Nonetheless, despite their offensive strategy and elevated latency before avoiding the predator, hamsters also increased their use of the APT when confronted with a mobile predator. Hence, this suggests that the offensive strategy used by hamsters is part of their risk assessment strategy. As indicated by Eibl-Eibestfeldt [18], the offensive strategy is especially important when a predator manages to closely approach the hamster (~2m). European hamsters usually mock the predator, but if the latter continues to approach, then hamsters attack and can even harm the predator by sinking their teeth into its legs.

The European hamster is one of the largest rodent in Europe, with adult males weighing up to 650g [23]. Hamsters possess long teeth and have often been described as very aggressive, especially females [15,18,23]. Therefore, similar to what has been observed in fish [10], the offensive strategy might be beneficial under some conditions for adults facing relatively small predators, such as the European ferret. Nonetheless, it is likely that the benefits of such strategy would be reduced when facing larger predators, such as foxes. Therefore, hamsters may use a different strategy towards such big predators [43]. Nonetheless, direct attacks against dogs and humans have been recorded under wild conditions [18] and several studies reported that aggressiveness is generally reduced in captive-reared versus wild individuals [44]. Given the importance of experience [6], it would be interesting to investigate the differences in behavioural responses of captive-reared and wild hamsters against predators bigger than cats and ferrets. Variations in ground cover should also be considered[45]. Moreover, it has been shown for several taxa that individuals are generally bolder, more explorative and more aggressive in highly anthropogenic environments, when compared with natural habitats [8,46,47]. Since European hamsters evolved in farmlands during the past centuries [13,23], but are now frequently found in urban areas [48], bold-reaction types might reflect an adaptation to habitat change that has been maintained under recent captive conditions. However, individuals that are more exploratory, bold and aggressive have reduced capacities to exploit new resources in changing or stochastic environments, when compared with shy individuals [49]. Indeed, the latter are more cautious and attentive to external stimuli and adapt better to changing environmental conditions [8,49,50]. Further research, studying captive-reared hamsters from different breeding units, as well as wild hamsters from urban areas and from farmlands is therefore required to better understand the environmental effects and fitness consequences of these bold behaviours. If possible, such studies should investigate the reactions to European hamsters to different predator and non-predator species, to more clearly determine the predator discrimination abilities of this endangered species.

## Conclusion

We have shown that captive-reared European hamsters displayed an offensive strategy towards the European ferret (no escaping, but mobbing behaviour and, in some cases, direct attack). Nonetheless, despite their bold behaviour, hamsters used the APT after perceiving an imminent risk of predation. Our study provides insights into the risk-assessment behaviour of captive-reared hamsters and also highlights inter-individual differences in their perception and reaction towards the urine and presence of known terrestrial predators. With respect to the APT, we have equipped and monitored several wildlife underpasses in the French distribution area of the European hamster to study its efficacy under wild conditions. This showed that while the APT improved the crossing frequency of male hamsters in the presence of a European ferret, this was not the case for females [51]. Nonetheless, investigation concerning the reactions of hamsters towards bigger mammalian (e.g. foxes) or avian (e.g. birds of prey) predators, also considering differences in ground cover, are urgently needed.

## Supporting information

S1 Table Data used to perform statistical analyses of the Y-maze test. (XLSX)

S2 Table Data used to perform statistical analyses of the experiment with a non-mobile ferret. (XLSX)

S3 Table Data used to perform statistical analyses of the experiment with a mobile ferret. (XLSX)
